# Neck Pain- and Unsteadiness-Inducing Activities and their Relationship to the Presence, Intensity, Frequency, and Disability of Headaches

**DOI:** 10.3390/brainsci10070425

**Published:** 2020-07-03

**Authors:** Daniel Rodríguez-Almagro, Alexander Achalandabaso-Ochoa, Francisco Javier Molina-Ortega, Esteban Obrero-Gaitán, Alfonso Javier Ibáñez-Vera, Rafael Lomas-Vega

**Affiliations:** Department of Health Science, University of Jaén, Paraje Las Lagunillas s/n, 23071 Jaén, Spain; dra00005@red.ujaen.es (D.R.-A.); fjmolina@ujaen.es (F.J.M.-O.); eobrero@ujaen.es (E.O.-G.); ajibanez@ujaen.es (A.J.I.-V.); rlomas@ujaen.es (R.L.-V.)

**Keywords:** disability, neck pain, unsteadiness, headache

## Abstract

(1) Background: Headache is a significant public health problem. Despite the association between headache and neck pain, little is known about the relationships among specific activities that generate neck pain and headache. The aim of this study was to identify the specific activities that result in neck pain and unsteadiness, and determine how they are linked to headache in university students. (2) Methods: One hundred and six patients with physician-diagnosed headache and 92 healthy university students completed surveys assessing demographics; the presence, frequency, intensity, and disability of headaches; and activities generating neck pain and unsteadiness. (3) Results: The presence of headache was related to female gender (*p* = 0.001), neck pain when reading or watching television (*p* = 0.024), and unsteadiness when moving the head (*p* = 0.005). Headache-related disability was associated with intensity of neck pain (*p* < 0.001), neck pain when reading or watching television (*p* = 0.033), and stumbling (*p* < 0.001). Headache frequency was related to smoking (*p* = 0.004), the duration of neck pain-associated symptoms (*p* = 0.047), and neck pain when driving (*p* = 0.039). Intensity of headache was associated with female gender (*p* = 0.002), smoking (*p* = 0.013), and neck pain-related sleep alterations (*p* = 0.024). (4) Conclusions: Female gender, smoking, neck pain, and unsteadiness when moving the head are factors related to headache in university students.

## 1. Introduction

Headaches are a significant public health problem in countries of all development levels, with higher prevalence among females, university students, and urban residents [1]. It is estimated that approximately 40.5% of the worldwide population was affected by migraine or tension-type headaches in 2016 [2]. Headaches have an important individual impact [3]. It has been observed that headache patients experience anxiety symptoms and condition their daily activities due to the fear of suffering a new headache attack, which leads to a reduction in useful time and productivity [3]. Headaches have an enormous economic and social impact, because of their high prevalence levels together with the elevated indirect cost associated with these disorders. In Europe, the total annual cost of headaches has been estimated at 173 billion euros [4]. 

The link between neck pain and headaches has been widely investigated, and a strong association between dysfunction in cervical structures and headaches has been demonstrated [5,6,7]. It has been observed that headache severity increases with as the prevalence of neck structure alterations increases [6]. Moreover, a strong association between these two factors has been reported in a young population [6], pointing to the important role that muscular pain plays in headache development among adolescents. Furthermore, both neck pain and headache have been linked to unsteadiness [8,9]. As a result, patients experience remarkable negative consequences in their social and professional lives [8], as headaches hinder their ability to concentrate and pay attention during several activities (whether work, study, or leisure). 

Moreover, lifestyle and other contextual factors (such as spending three or more hours sitting) can augment the prevalence of both musculoskeletal complains and headache [10] due to maladaptive postures that increase the craniocervical load [11]. Furthermore, the young population also appears to be especially vulnerable to electronic device exposure, given the enormous time spent using these devices in their daily routines. These habits are especially considered a risk factor for the development of migraines [12]. The frequent use of electronic devices (tables, telephones, etc.) can increase the mechanical load on the cervical spine, which may increase the compressive forces on the cervical apophyseal joints and the mechanical stress on the shoulders and neck muscles, thereby sensitizing both the cervical spine [13] and the trigeminal–cervical complex, which may initiate the headache process [14,15].

Despite the connection between headache, neck pain, and unsteadiness, little is known about which kinds of neck-pain-generating activities or unsteadiness-inducing activities may cause headaches and how they could contribute to headaches. Finding out which activities can trigger headaches may facilitate their assessment and treatment. For these reasons, the present study intends to identify which activities generate neck pain or unsteadiness and how they are related to the presence, frequency, intensity, and disability of headaches in university students.

## 2. Materials and Methods

### 2.1. Study Design

A cross-sectional study was designed and implemented according to the guidelines for the communication of observational studies established in the Strengthening the Reporting of Observational Studies in Epidemiology (STROBE) guide [16]. This study was carried out in accordance with the Helsinki Declaration, good clinical practices, and all applicable laws and regulations and was approved by the Ethics Committee of the University of Jaén (reference number ABR 7/17). Patients who met the eligibility criteria gave informed consent and completed questionnaires.

### 2.2. Participants

Contact with participants was established at the Health Sciences Faculty of the University of Jaén (Spain) through posters and digital announcements. All participants were young adult undergraduate and graduate students over 18 years of age. In January 2017, 253 students were screened to participate in the present study. After being duly informed about its specifics, 211 students agreed to participate, of which 198 subjects completed all questionnaires and evaluations. Data collection was carried out between February and April 2017 at the University of Jaén. To participate in the study, it was necessary to be a university student and to sign the informed consent form. Exclusion criteria were the presence of cognitive disturbance; ocular disease; previous head or neck trauma; any type of acquired brain damage (ischemic or hemorrhagic stroke or damage derived from intracranial intervention); any systemic disease with visual, vestibular, central, or musculoskeletal affectation; neuromuscular disease; or neoplasia at the visual, vestibular, or central level.

A physician (F.H.) classified the participants into the headache or non-headache categories following the criteria described in the third edition of the *International Classification of Headache Disorders* [17] at the time of inclusion.

**Figure 1:**
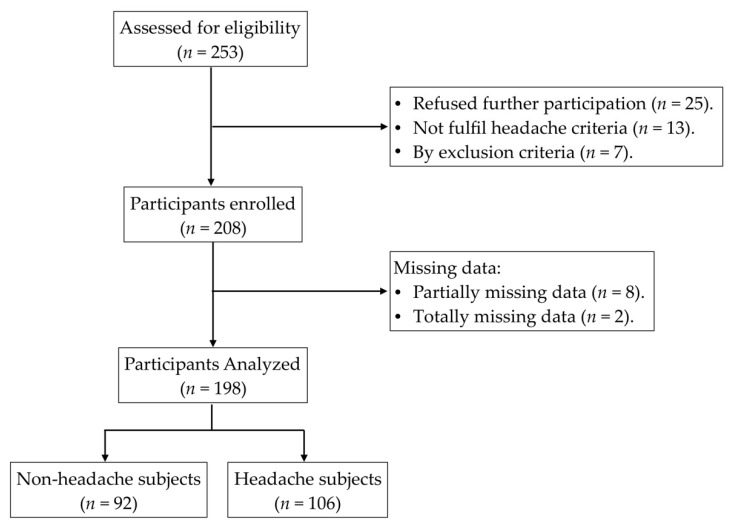
Flowchart of participant selection through the study.

The selection process of participants is graphically represented in Figure 1.

### 2.3. Measurements

Before completing the questionnaires, the participants reported their sociodemographic data including gender, age, height, weight, which year of college they were in, smoking habits, and physical activity.

The Spanish version of the Northwick Park Questionnaire [18] was used to obtain the variables related to the activities of daily life that generate neck pain. The Northwick Park Questionnaire is a self-administered questionnaire of 10 items with moderate total test–retest reliability values (Intraclass Correlation Coefficient (ICC) = 0.63) and moderate to high values of reliability (Intraclass Correlation CoefficientICC = 0.42 to 0.85). The construct validity of the Northwick Park Questionnaire with respect to the Visual Analogue Scale showed a medium-size correlation value for the initial measurement (r = 0.51, *p* < 0.001) and a strong correlation value for the retest measurement (r = 0.74, *p* < 0.001) [18]. Although the Northwick Park Questionnaire provides information on how neck pain has affected patients’ ability to manage in everyday life, some of its items address the generation of neck pain during the performance of certain activities. An example is Item 6, which asks about neck pain when reading and watching television, with one of the response options being “I can do this as long as I wish, but it causes extra pain”.

The unsteadiness questionnaire developed by Renaud et al. [19] was used to obtain the variables related to symptoms of unsteadiness. This self-implemented questionnaire provides a standardized report on the symptoms of unsteadiness in specific situations. The questionnaire’s cut-off point is four, with 100% sensitivity and 98% specificity to discriminate between subjects with unsteadiness and healthy subjects. In addition, this questionnaire presents good reliability results (KR-20 = 0.75). The questionnaire consists of nine items that allow a dichotomous answer (yes/no) [19]. The items are related to specific situations in which symptoms of unsteadiness are revealed. As additional information, in three out of the nine items, patients are required to specify the frequency and time since the symptoms last occurred.

The presence/absence of headache was determined through the medical diagnosis made at the beginning of the present study. The degree of headache-related disability, as a way to estimate the personal impact of headache in terms of lost useful time, as well as the frequency and intensity of headache—two of the most important criteria in headache diagnosis—were estimated through the Spanish version of the Migraine Disability Assessment questionnaire [20]. Seven items compose this instrument: the first five items focus on three dimensions of daily life that can be affected by headaches, while the other two items refer to the frequency and intensity of headaches. The sum of the score of the first five items provides the degree of headache-related disability, while the sixth and seventh items indicate the frequency and intensity of headaches, respectively. This questionnaire presents excellent reliability values for the main scale (ICC = 0.81; 95% C.I. = 0.63–0.90; *p* < 0.001) and for headache frequency (ICC = 0.90; 95% C.I. = 0.79–0.95; *p* < 0.001), and good for headache intensity (ICC = 0.63; 95% C.I. = 0.34–0.80; *p* < 0.001) [20].

### 2.4. Sample Size Calculation

For sample size calculation, we required at least 10 observations per independent variable included in the multiple linear regression model [21], as well as at least 10 subjects per event in the multiple logistic regression model [22]. Given that the linear regression models employed a maximum of 9 variables (obtained in the bivariate analysis), for each predicted dependent variable related to headache (presence, intensity, frequency, and disability) and with an estimated prevalence of headache around 55%, 190 subjects were required for the purposes of our analysis.

### 2.5. Statistical Analysis

The categorical variables were described using frequencies and percentages, while the continuous variables were described using means and standard deviations. Each item of the Northwick Park Questionnaire and the unsteadiness questionnaire, as well as the sociodemographic parameters, were considered to be independent variables, while the presence/absence of headache, the level of headache-related disability, and the intensity and frequency of headache were considered to be dependent variables. Given that the items of the Northwick Park Questionnaire have an ordinal-type response, the responses of the participants were recoded as dichotomous, where 0 indicated “no neck pain generated” and 1 indicated “some neck pain generated”.

The identification of the factors related to the presence/absence of headache was performed using a multiple logistic regression model due to the dichotomous nature of the dependent variable. The analysis included the entire sample, and the relationships between the presence of headaches and the independent variables were analyzed using simple logistic regression. The variables that presented a statistically significant odds ratio (*p* < 0.05) were selected for the multiple logistic model. A forward stepwise selection (conditional) method was used to add the independent variables to the multiple logistic model.

Prediction of continuous variables (intensity, frequency, and disability of headache) was performed using only the data from the headache subjects. The relationships among each of the independent and dependent variables were explored by simple linear regression. The dependent variables were the intensity, frequency, and disability of headache. The factors related to the dependent variables were identified using a multiple linear regression model specific to each variable. The independent variables that presented a statistically significant β value (*p* < 0.05) were entered in each multiple linear model. A forward stepwise selection method was used to add the variables to the different multiple models.

The multivariate coefficient of determination (R^2^) was used to calculate the effect size in the linear regression models, whereas Nagelkerke’s R^2^ was used to calculate the effect size in the logistic regression model. According to Cohen, R^2^ can be considered insignificant when it is below 0.02, small when it is between 0.02 and 0.15, medium when it is between 0.15 and 0.35, and large when it is above 0.35 [23]. The associations between the variables related to neck pain and unsteadiness were also analyzed. Potential collinearity was verified by excluding the variables that presented a variance inflation factor above 10 [24]. We set the level of statistical significance at *p* < 0.05. Data management and analysis were performed using IBM SPSS Statistics 23.0 for Windows (SPSS Inc., Chicago, IL, USA).

## 3. Results

Initially, 253 subjects were invited to participate in the study, out of which 211 were enrolled and 198 completed the study. Of these, 40.9% were men and 59.1% were women. Ninety-two subjects were classified as healthy subjects and 106 were classified as headache subjects, according to the criteria of the *International Classification of Headache Disorders* published in 2013 [17]. The global prevalence of headaches was 53.5%. In the headache group, 25.5% were men and 74.5% were women. Greater values of frequency and intensity of headache, as well as a higher headache-related disability level were observed in the headache group. Table 1 shows the sociodemographic data of the participants.

The multiple logistic regression model used to identify the factors related to the presence of headache showed that the predictor variables were gender, neck pain when reading or watching television, and the presence of unsteadiness when moving the head (Table 2). The multiple model predicted 23.1% of the variance of the dependent variable (R^2^ = 0.231, *p* < 0.001).

The multiple linear regression model used to identify the factors related to headache-related disability showed that the predictor variables were intensity of neck pain, neck pain when reading or watching television, and stumbling (Table 3). The multiple model predicted 31% of the variance of the dependent variable (R^2^ = 0.309, *p* < 0.001).

The multiple linear regression model used to identify the factors related to frequency of headache showed that the predictor variables were the duration of neck pain-associated symptoms, smoking, and neck pain when driving (Table 4). The multiple model predicted 22.7% of the variance of the dependent variable (R^2^ = 0.227, *p* = 0.039).

The multiple linear regression model used to identify the factors related to the intensity of headache showed that the predictive variables were gender, smoking, and neck pain-related sleep alterations (Table 5). The multiple model predicted 19% of the variance of the dependent variable (R^2^ = 0.190, *p* < 0.001).

## 4. Discussion

The major finding of this cross-sectional study is the existence of a concomitant relationship among neck pain-inducing activities, unsteadiness-inducing activities, and headache in university students. Significant associations were found for headache with several factors such as neck pain when performing high attention-demanding activities (reading, watching television, or driving); the presence of unsteadiness when moving the head; stumbling during walking; and other sociodemographic factors, among which smoking as an habit and the female gender were emphasized.

The link between neck pain and headache has been amply demonstrated [5,6,7]. In our study, we observed that neck pain intensity and neck pain-associated symptoms are linked to increased levels of headache-related disability and headache frequency, respectively. In light of these relationships, it is possible to conclude that there is an association between neck pain and headache severity, as previously described by other authors [6]. In this regard, Blake and Burstein showed that the simultaneous presence of both neck pain and headache may be caused by the compression of the lesser and greater occipital nerves by posterior cervical muscles and their fascial attachments at the occipital ridge with subsequent local perineural inflammation [25].

Moreover, we found that high visual attention-demanding activities that produce neck pain, such as reading, watching TV, or driving, are linked to the presence of headache, higher headache-related disability levels, and to a gain in headache frequency. It has been observed that continuous visual efforts maintained under inappropriate conditions (exposure to visual displays, excessive proximity to the displayed object, poor lighting) [26] may induce deficits in oculomotor control, such as a reduction in the speed of smooth pursuit and saccadic eye movements, which are related to dysfunction in the upper cervical structures [27]. In turn, these may cause headaches by the convergence of cervical and trigeminal afferent fibers at the trigeminal–cervical complex [15].

A link was previously found between unsteadiness and headaches [8,9], and balance alterations have been shown to have a great impact on headache-related disability [8]. In agreement with these previous findings, we were able to establish relationships between unsteadiness-inducing fast head movements and headaches, and between stumbling (as an indicator of balance alterations) and increased headache-related disability. A link between the trigeminal nucleus and vestibular nuclei may be responsible for these relationships. This link has been demonstrated in rats [28]. In addition, it has been suggested that an imbalance of the vestibular system may be induced by a painful trigeminal stimulation in migraine patients [29], and some authors have proposed that vestibular disorder should be considered an integral manifestation of headache and not as two different and concomitant diseases [30].

It seems that trigeminal nuclei are not only related to neck pain but also to vestibular disorders in relation to headache development. Thus, the impact of neck pain- and unsteadiness-inducing factors on the presence and development of headaches may have a common origin in the influence of the trigeminal complex. Therefore, the presence of one of the three factors may lead to the apparition of the other two, as a consequence of the sensitization process of this complex, which is common to the three maladies [15,29].

Sleep disturbances are not only common symptoms among patients with neck pain [31] but seem to play an important role in headache development. With regard to both neck pain and headaches, it has been possible to establish a link between poor sleep quality due to neck pain-associated symptoms and higher levels of headache intensity, which is in accordance with previous findings [32]. Previously, in patients with headaches, it has been reported that headache attacks are preceded by a lack of sleep [33,34], and sleep is considered to have a therapeutic effect on headache management [34].

In addition, we have found that smoking is linked to increased headache frequency and intensity, which is in agreement with Taylor [35] and Rozen [36], who proposed smoke as a chronification factor. Furthermore, we observed gender to be related to headaches and higher headache intensity. Women’s relative risk of suffering from headaches is around twice as high as that of men, with a prevalence of 68% for the former and 33% for the latter. This could be related to the higher prevalence of neck pain in the female population [37] and the anatomical differences between men and women [38].

The present study has several limitations. First, the study was conducted on a population from a very concrete geographical area and a very specific age group, and any generalization of its results should be limited to individuals with similar characteristics. Therefore, our conclusions cannot be extrapolated to other population groups. Second, the cross-sectional methodology used in this study does not allow causal relations to be drawn.

Despite these limitations, to the best of our knowledge, this is the first study to investigate factors contributing to headaches among university students. Future research could look into the influence of the overuse of electronic devices on the social and academic performance of students. At the same time, and given the relevance that dysfunctions of the upper cervical structures have in this pathology, it would be interesting to explore headache management through the treatment of cervical structures among university students, identifying which activities have a great influence on the headache process, addressing them, and acting over the structures affected, as well as investigating possible secondary impacts on headache development, possibly at the expense of often unnecessary systematic pharmacological treatment [39].

## 5. Conclusions

In summary, the presence of headaches in university students is related to female gender, to neck pain-generating activities such as reading or watching television, and to unsteadiness when moving the head. Headache-related disability is associated with the intensity of neck pain, neck pain when reading or watching television, and unsteadiness, leading to stumbling. The frequency of headaches is related to smoking, the duration of neck pain-associated symptoms, and neck pain when driving. Finally, the intensity of headache is associated with female gender, smoking, and neck pain-related sleep alterations.

## Figures and Tables

**Table 1:** Description of the study participants.

		Headache (*n* = 106)		Non-Headache (*n* = 92)		Total (*n* = 198)	
Categorical		F	%	F	%	F	%
Gender	Male	27	25.5	54	58.7	81	40.9
	Female	79	74.5	38	41.3	117	59.1
Course	First course	36	34.0	25	27.2	61	30.8
	Second course	37	34.9	31	33.7	68	34.3
	Third course	9	8.5	16	17.4	25	12.6
	Fourth course	12	11.3	6	6.5	18	9.1
	Master	12	11.3	14	15.2	26	13.1
**Continuous**		**Mean**	**SD**	**Mean**	**SD**	**Mean**	**SD**
Age (years)		21.20	2.80	21.69	3.32	21.43	3.05
Height (cm)		168.08	8.08	172.38	9.51	170.08	9.01
Weight (kg)		64.04	11.22	69.88	13.92	66.75	12.85
BMI		22.55	2.97	23.30	3.16	22.90	3.07
MIDAS		5.01	9.05	0.80	2.43	3.06	7.13
Headache frequency ^a^		5.79	6.84	0.79	1.35	3.47	5.66
Headache intensity (0/10) ^a^		4.10	2.26	1.60	2.10	2.94	2.51

**^a^** Mean value of the last three months. Abbreviations. MIDAS: Migraine Disability Assessment Questionnaire; BMI: Body Mass Index; F: Frequency. SD: Typical Deviation.

**Table 2:** Univariate and multivariate logistic regression to analyze the factors related to the presence of headache.

	Univariate Analysis				Multivariate Analysis			
Variable	OR	95% C.I.		*p*	OR	95% C.I.		*p*
		Inferior	Superior			Inferior	Superior	
Gender	4.158	2.276	7.596	<0.001	3.029	1.597	5.746	0.001
Height (cm)	0.946	0.915	0.978	0.001	NS	NS	NS	NS
Neck pain intensity	2.782	1.517	5.102	0.001	NS	NS	NS	NS
Neck pain-associated symptoms duration	3.148	1.515	6.540	0.002	NS	NS	NS	NS
Neck pain when reading or watching TV	2.582	1.454	4.586	0.001	2.042	1.100	3.792	0.024
Unsteadiness when moving the head	3.640	1.998	6.631	<0.001	2.544	1.335	4.851	0.005
Unsteadiness during fast position changes	2.533	1.407	4.560	0.002	NS	NS	NS	NS

Abbreviations. OR: Odds Ratio; 95% C.I.; 95% Confidence Interval; NS: Non-Significant.

**Table 3:** Univariate and multivariate lineal regression to analyze the factors related to headache-related disability.

	Univariate Analysis				Multivariate Analysis			
Variable	B	95% C.I.		*p*	B	95% C.I.		*p*
		Inferior	Superior			Inferior	Superior	
Neck pain intensity	3.709	1.952	5.466	<0.001	3.308	1.671	4.945	<0.001
Neck pain-related sleep disturbances	3.607	1.306	5.908	0.002	NS	NS	NS	NS
Duration of neck pain-associated symptoms	2.819	1.136	4.502	0.001	NS	NS	NS	NS
Neck pain when lifting weights	4.878	0.900	8.856	0.017	NS	NS	NS	NS
Neck pain when reading or watching TV	2.652	0.704	4.601	0.008	1.895	0.158	3.632	0.033
Neck pain at work	4.751	1.467	8.034	0.005	NS	NS	NS	NS
Neck pain at driving	4.873	0.829	8.917	0.019	NS	NS	NS	NS
Stumbling	3.654	1.838	5.471	<0.001	3.672	2.034	5.310	<0.001

Abbreviations. B: Beta value; 95% C.I.; 95% Confidence Interval; NS: Non-Significant.

**Table 4:** Univariate and multivariate lineal regression to analyze the factors related to frequency of headache.

	Univariate Analysis				Multivariate Analysis			
Variable	B	95% C.I.		*p*	B	95% C.I.		*p*
		Inferior	Superior			Inferior	Superior	
Smoking	6.926	3.154	10.705	<0.001	5.445	1.729	9.16	0.004
Neck pain intensity	2.279	0.913	3.645	0.001	NS	NS	NS	NS
Duration of neck pain-associated symptoms	2.534	1.289	3.779	<0.001	1.399	0.016	2.781	0.047
Neck pain when lifting weights	4.454	1.485	7.424	0.004	NS	NS	NS	NS
Neck pain when reading or watching TV	1.815	0.332	3.298	0.017	NS	NS	NS	NS
Neck pain at work	4.061	1.605	6.517	0.001	NS	NS	NS	NS
Neck pain when driving	5.062	2.078	8.045	0.001	3.284	0.162	6.407	0.039

Abbreviations. B: Beta value; 95% C.I.; 95% Confidence Interval; NS: Non-Significant.

**Table 5:** Univariate and multivariate lineal regression to analyze the factors related to the intensity of headache.

	Univariate Analysis				Multivariate Analysis			
Variable	B	95% C.I.		*p*	B	95% C.I.		*p*
		Inferior	Superior			Inferior	Superior	
Gender	1.481	0.520	2.442	0.003	1.471	0.558	2.385	0.002
Smoking	1.763	0.485	3.042	0.007	1.505	0.318	2.692	0.013
Neck pain intensity	0.497	0.033	0.961	0.036	NS	NS	NS	NS
Neck pain-related sleep disturbances	0.741	0.130	1.299	0.017	0.640	0.088	1.192	0.024
Duration of neck pain-associated symptoms	0.651	0.228	1.075	0.003	NS	NS	NS	NS
Neck pain when reading or watching TV	0.580	0.089	1.070	0.021	NS	NS	NS	NS
Neck pain at work	0.959	0.128	1.790	0.024	NS	NS	NS	NS
Unsteadiness when moving head	1.072	0.087	2.057	0.033	NS	NS	NS	NS
Dizziness	1.000	0.128	1.872	0.025	NS	NS	NS	NS

Abbreviations. B: Beta value; 95% C.I.; 95% Confidence Interval; NS: Non-Significant.
